# CAR-T cells leave the comfort zone: current and future applications beyond cancer

**DOI:** 10.1093/immadv/ltaa006

**Published:** 2020-11-25

**Authors:** Mariana Torres Mazzi, Karina Lôbo Hajdu, Priscila Rafaela Ribeiro, Martín Hernán Bonamino

**Affiliations:** Immunology and Tumor Biology Program - Research Coordination, Brazilian National Cancer Institute (INCA), Rio de Janeiro, Brazil; Immunology and Tumor Biology Program - Research Coordination, Brazilian National Cancer Institute (INCA), Rio de Janeiro, Brazil; Immunology and Tumor Biology Program - Research Coordination, Brazilian National Cancer Institute (INCA), Rio de Janeiro, Brazil; Immunology and Tumor Biology Program - Research Coordination, Brazilian National Cancer Institute (INCA), Rio de Janeiro, Brazil; Vice - Presidency of Research and Biological Collections (VPPCB), Oswaldo Cruz Foundation (FIOCRUZ), Rio de Janeiro, Brazil

**Keywords:** chimeric antigen receptor, autoimmune diseases, infectious diseases, fibrosis, CAR-T cell, adoptive cell transfer

## Abstract

Chimeric antigen receptor (CAR)-T cell therapy represents a breakthrough in the immunotherapy field and has achieved great success following its approval in 2017 for the treatment of B cell malignancies. While CAR-T cells are mostly applied as anti-tumor therapy in the present, their initial concept was aimed at a more general purpose of targeting membrane antigens, thus translating in many potential applications. Since then, several studies have assessed the use of CAR-T cells toward non-malignant pathologies such as autoimmune diseases, infectious diseases and, more recently, cardiac fibrosis, and cellular senescence. In this review, we present the main findings and implications of CAR-based therapies for non-malignant conditions.

## Introduction

Immunotherapy has been a paradigm change in cancer treatment. The first documented attempts to use an immune-based therapy to treat cancer date back to William Coley in the 1890s, who used bacteria extracts injections directly into his patients’ tumors [1]. Although the data generated in those experiments are hard to interpret, the main idea of immunostimulation as cancer therapy has been carried on.

Bone marrow transplantation, the oldest form of cell-based therapy, was pioneered by the work of E. Donnall Thomas in the 1950s. He established the basic concepts that allowed for the first successful bone marrow transplantation in humans, a procedure that remains, up to now, the only curative option for a variety of hematological malignancies.

Since the establishment of bone marrow transplants, much has been achieved regarding immune-based therapy, whether humoral or cellular.

**Figure 1.**
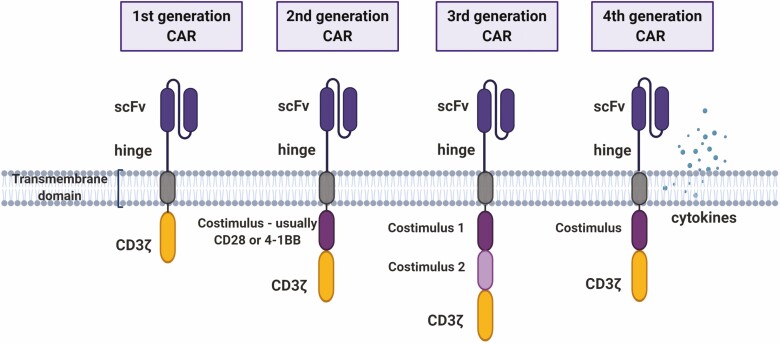
CAR generations. First-generation CARs comprised a single CD3zeta chain, while second-generation CARs included a costimulatory signaling domain. Later, third-generation CARs emerged combining two different costimulatory molecules, the most common being 4-1BB and CD28. Further developments, comprising fourth-generation CARs, included the expression or secretion of additional molecules to improve CAR-T cell performance.

The first monoclonal antibody was approved for B cell lymphoma in 1997 [2], and subsequently many others have been licensed for different malignancies. Later, antibodies were conjugated to chemotherapy agents in order to enhance potency and target specificity [3]. Checkpoint inhibitors [4] and bi-specific antibodies [5], which can play different roles such as engaging the host immunity against a tumor target, have also reached clinical use more recently. However, the most striking progress has been the approval in 2017 of chimeric antigen receptor (CAR)-T cells to treat B cell malignancies [6]. Considered the first ‘living drug’ to reach the market, CAR-T cells comprise T lymphocytes genetically engineered to express a CAR. The most common CAR configuration consists of an extracellular single-chain variable fragment (scFv) domain for major histocompatibility complex (MHC)-unrestricted target recognition, coupled to the transmembrane and intracellular signaling regions (Fig. 1). First-generation CARs were initially proposed in 1993 [7] and had a single CD3 zeta chain intracellular domain, which is able to replicate the T cell receptor (TCR)-derived signal 1 required for T cell activation. Second-generation CARs incorporated a costimulatory domain, usually CD28 or 41BB, while third-generation CARs have two costimulatory domains in tandem [8]. Additional developments, comprising the so-called fourth-generation CARs, included the expression or secretion of additional molecules like cytokines, to improve efficiency [9]. Even though CAR-T cells have reached their epitome in the treatment of B cells malignancies, the technology was not initially designed solely for this purpose and took advantage of many concepts developed during the study of other disease entities, such as human immunodeficiency virus (HIV) [10]. Notably, CAR-T cells clinical success in the cancer setting has opened a new horizon of possibilities, not only for cancer treatment, but also for a variety of diseases in which specific membrane antigens become expressed or up-regulated in pathological cells. These cells can be immunologically targeted and as so, could benefit from CAR-T cell therapy, especially chronic diseases for which immunological memory may provide long-term gain. A landscape covering different approaches of CAR-T development for non-malignant pathologies will be further described here.

## CAR-T cells in infectious diseases

### CAR-T cell development for HIV: past and present

**Figure 2.**
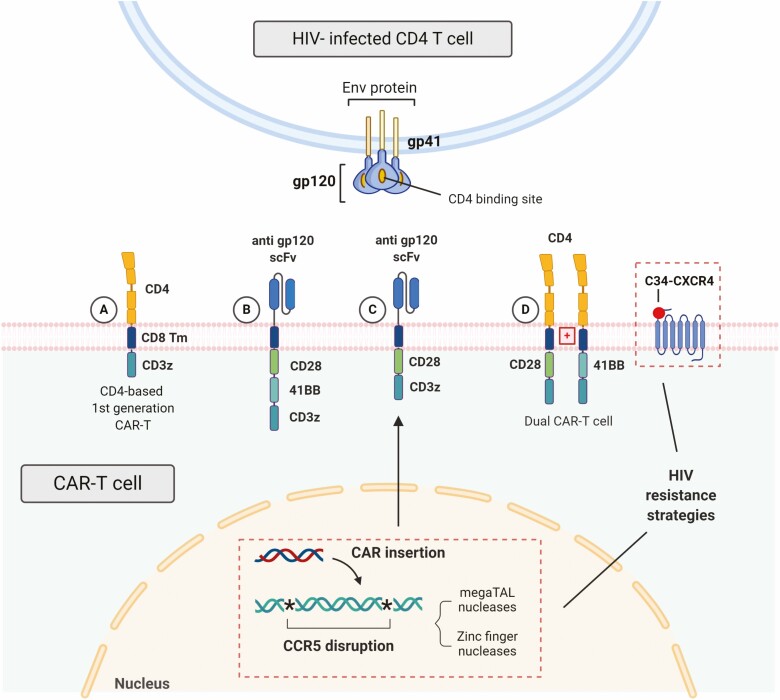
Past and present approaches for anti-HIV CAR-T cells. (A) The first-generation CAR construct used CD4 as the extracellular domain, coupled with a CD8 transmembrane region and a CD3zeta chain intracellular signaling. This structure rendered the CAR-T cell susceptible to HIV infection and showed limited viral control. To improve HIV infection resistance and persistence, subsequent designs have been optimized. (B) A third-generation CAR with the scFv fraction based on a broadly neutralizing antibody (bNAb) against gp120 and two costimulatory domains (CD28 and 4-1BB) is currently undergoing clinical investigation. (C) A second-generation CAR also comprising a scFv based on anti-gp120 bNAb with a single CD28 costimulatory domain, is expressed after CCR5 disruption using megaTAL nucleases or zinc-finger nucleases, and incorporation of the donor template in its site. This strategy protects against insertional mutagenesis and has been proven safe in a clinical trial. (D) Co-expression of the CAR and inhibitory molecules has also been described. On (D), a dual-CAR-T cell co-expresses two CAR constructs independently along with the CXCR4-CD34 fusion inhibitor. The CD4 extracellular is common to both receptors, which differ only in the costimulatory domain (4-1BB or CD28). In a humanized BLT mouse model, the dual CAR has shown better viremia control compared to the separate constructs or a third-generation CAR with both co-stimulatory domains.

HIV and the associated acute immunodeficiency syndrome were described in the beginning of the 1980s and rapidly spread throughout the world. The significant human and economic burden associated with the disease-generated massive research efforts toward an efficacious treatment. Many drugs were developed, and even though the combination antiretroviral therapy (cART) is highly effective in controlling viral replication, it fails to eliminate the virus, and viremia recrudescence from the latent reservoir is observed upon treatment interruption [11]. The first reports of CAR-T cells clinical use date back to the 1990s, as an attempt to cure HIV. Those first-generation CARs comprised a CD4 extracellular domain associated to the CD3ζ (CD4ζCAR) signaling domain, which were transduced into CD8+ T cells (Fig. 2A). Clinical studies showed that while the strategy was feasible and safe, it failed to consistently control HIV replication [10, 12–15]. Nonetheless, one of the pioneering trials provided valuable information on CAR-T cell persistence, with decay half-lives exceeding 16 years [16]. Since then, CAR-T cell design evolved and key factors, such as effector function, persistence, and susceptibility to exhaustion, have been greatly optimized (reviewed in Chicaybam *et al*. [17]). Although second-generation CARs show better *in vitro* activity against HIV and *in vivo* persistence, this has never translated into clinical benefit [18]. Several challenges remain, such as the optimal extracellular target, long-term persistence, and the ability to target the latent viral reservoir.

HIV is a highly mutating virus and a pivotal point on cellular invasion is the recognition of CD4, mediated by the viral envelope protein (Env) gp120 binding. Because this domain is a key target, CD4 poses an attractive extracellular CAR domain, as the binding site of gp120 tends to remain conserved. However, CD4 expression also renders the CAR-T cells susceptible to lytic infection and destruction. In fact, the clinical studies did not show any beneficial role of these first-generation CARs with CD4 and the zeta chain. CARs with scFv derived from broadly neutralizing antibodies (bNAbs) that target conserved sites in the Env protein have also been developed [19–21]. One of them, a third-generation CAR with the scFv derived from a Nab directed against gp120, VRC01 [21], is undergoing clinical trial (NCT03240328) (Fig. 2B).

Protection of CAR-T cells from HIV infection is also paramount for therapy success and several approaches have tried to prevent HIV entrance in the cells [18]. CAR-T cells with targeted disruption of the HIV co-receptor CCR5 using zinc finger nucleases have been developed [22, 23] and are currently undergoing clinical investigation (NCT03617198). Recently, a promising new CAR engineering strategy enabling high rates of homology-directed repair (HDR) has been described. In this study, CCR5 was disrupted using megaTAL nucleases, and HDR allowed for simultaneous incorporation of the donor template into the CCR5 site (Fig. 2C). The resulting CAR-T cells showed better inhibition of HIV *in vitro* compared to non-CCR5 disrupted cells, and also added a potential new safety tool against insertional mutagenesis [20, 24]. This strategy was proved safe in a clinical trial [25]. Co-expression of CAR and inhibitory molecules, such as C34-CXCR4, an HIV fusion inhibitor, has also been described [26]. In a recent approach, Maldini *et al*. developed a dual CD4-based CAR-T cell that expresses both 41BB- and CD28- second-generation CARs independently, along with the C34-CXCR4 fusion inhibitor [27] (Fig. 2D). This construct resulted in superior proliferative capacity and persistence in a humanized BLT (bone marrow, liver, and thymus) mouse model when compared with the separate second-generation CARs or with a third-generation construct comprising both 41BB and CD28 costimulatory domains. The dual CAR expressing the fusion inhibitor led to reduction in acute viremia *in vivo* and enhanced suppression of viral load in a combined treatment with cART compared with cART alone.

Yet another way of targeting poor persistence of CAR-T cells *in vivo* was the generation of CD34+ hematopoietic stem and progenitor cells (HSPC)-derived CAR-T. In an immunodeficient murine model, CD4ζCAR was transduced into human CD34+ HSPC, generating T and B lymphocytes, NK cells, and myeloid cells in the animals. The authors showed that CAR-expressing cells were present in the thymus, spleen, blood, and bone marrow. Upon HIV challenge, CAR-T cells proliferated and were seemingly better at suppressing viral load than controls [28, 29]. However, a subsequent study with non-human primates failed to show a significant effect on viremia in the absence of cART, even though the cells persisted for as long as 2 years [30].

HIV-targeted CAR-T cells must also overcome the low antigen burden, which poses a challenge for long-term persistence. Preceding CAR-T infusion with TCR-specific viral antigen immunization is a way to address this issue and has already been included in clinical trials for B cell malignancies and glioblastoma (NCT01430390, NCT00709033, NCT01109095, and NCT03186118). Double-specific CAR-T cells are manufactured using cytomegalovirus (CMV), Epstein–Barr virus (EBV), or adenovirus TCR-specific cells [31–33] and after infusion, vaccination with viral epitopes is able to elicit CAR-T proliferation and enhance antitumor activity. However, it may prove counterproductive, as repetitive TCR activation leads to terminal differentiation and diminished proliferative capacity. The fine-tuning of stimulation frequency and intensity is yet another challenge for this approach.

Despite the high efficacy of cART, HIV elimination has not been achieved due to the presence of a latent virus reservoir [34]. The ‘shock and kill’ strategy with latency-reversing agents (LRAs) aimed at reversing quiescence to reestablish immune surveillance, also failed to destroy the reservoir [35–38]. The main sites of HIV persistence are CD4 T follicular helper cells (T_FH_) in B cell follicles [39]. Cytotoxic T lymphocytes (CTLs) are incapable of reaching these follicles because they lack CX chemokine receptor 5 (CXCR5), which guides cell trafficking in the B cell zone through a CXC chemokine ligand 13 (CXCL13) gradient [39]. Co-expression of CAR and CXCR5 is a reasonable approach that has been described in a simian model. As a proof of principle, the SIV-targeted CAR-CXCR5 cells showed *in vitro* migration through a CXCL13 gradient, and *ex vivo* homing to B follicles [40]. The combination of a CAR-T and LRAs is also being investigated in a clinical trial (NCT03980691).

### CAR T cells in other infectious diseases

**Figure 3.**
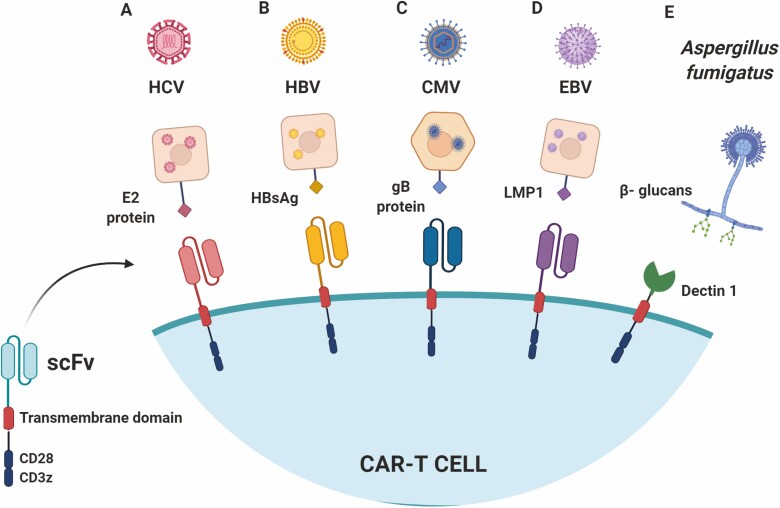
Current development of CAR-T cells for infectious diseases beyond HIV. (A). Second-generation CARs were designed with scFv domains anti-HCV (A) and HBV(B) antigens. Other CARs were designed to recognize epitopes of proteins found on the membrane of the target cells which are essential for virus entrance, as the gB protein in CMV infection (C) and LMP1 in EBV infection (D). (E) Second-generation CAR using the Dectin-1 protein extracellular portion, a natural receptor of B-glucans which are present in *Aspergillus sp.* cell wall.

Beyond HIV, CARs specific for hepatitis C virus (HCV) have recently been developed based on an scFv targeting a highly conserved epitope of the E2 glycoprotein present in the HCV viral capsid. Sautto *et al*. [41, 42] evaluated the activity of anti-HCV CAR-T cells *in vitro* and were able to induce lysis of target cells transfected with HCV E2 (Fig. 3A). However, this anti-HCV CAR-T has not yet been tested *in vivo*. Because the structural properties of the E2 glycoprotein have not been well established, further studies are necessary to expand this approach.

In other models, such as for hepatitis B infections (HBV), some CARs have also been developed to prevent hepatocellular carcinoma progression and to approach chronic diseases. In this last example, the sequences of proteins S and L, both present on the surface of HBV infected cells, are used as targets for scFvs (Fig. 3B). These CARs are engineered to be expressed in lymphocytes from healthy donors. In this study, Bohne *et al*. [43] observed that anti-HBV CAR-T cells were able to recognize hepatocytes infected by HBV *in vitro* producing IL-2 and IFN-g following targeted recognition. It was also possible to observe that the production of these cytokines occurred at earlier time points in S-specific CAR-T cells than in L-specific cells, probably due to the high expression of protein S on the surface of infected cells.

Kruse *et al*. [44] also created CARs to recognize other HBV antigens by using different sizes of spacers in the structure of CARs. The authors observed that CARs that contained larger spacers were more promptly activated, producing higher levels of pro-inflammatory cytokines such as TNF-a, IL-2, and IFN-g when co-cultured with different cell lines infected by HBV *in vitro*. Subsequently, it was observed *in vivo* that after HBV infection and subsequent anti-HBV CAR-T treatment, there was a reduction in plasmatic HBV antigens and DNA, although it was not possible to observe complete elimination of the virus. However, the use of anti-HBV CAR-T cells is still more promising than the use of the anti-HBV therapies already known and currently used, like the nucleoside analogues mentioned previously. This aspect is particularly interesting because these analogs block the synthesis of viral DNA but do not affect the production of antigens. Thus, hepatocytes infected by HBV, even under treatment with nucleoside analogs, can still be targeted by anti-HBV CAR-T cells [45].

The use of CARs has also been evaluated in models of CMV infection. Anti-gb CAR-T cells, which target a protein expressed in the viral capsid, were successfully activated after co-culture with CMV-infected cells, in addition to showing satisfactory production of TNF-a and IFN-g [46] (Fig. 3C). However, in 2016, Proff *et al*. [47] noted that CMV-infected cells were resistant to death by anti-CMV CAR-T cells. Subsequently, it was possible to verify that some anti-apoptotic viral factors, which can inhibit the death of infected host cells, contribute to dampening the cytotoxicity of T cells. The same was observed when anti-CMV CAR-T cells were co-cultured with fibroblasts not infected by CMV, but with overexpression of the UL37x1 and UL36 proteins, showing that in addition to the deficient antigen presentation, these viral proteins protect the infected cells, blocking the cytotoxic effector functions of anti-CMV CAR-T cells. These observations do not exclude the potential for the use of antiviral CAR-T cells, since T lymphocytes remain capable of exerting non-cytotoxic functions through the secretion of cytokines and granzymes, which are important for cleavage of essential proteins related to CMV replication [48].

CARs have also been developed to recognize EBV proteins. In one of these approaches, Tang *et al*. [49] used a CAR specific for EBV latent membrane protein 1 (LMP1) (Fig. 3D). The CAR-T cells were co-cultured with nasopharyngeal carcinoma cells overexpressing LMP1, resulting in activation and production of cytokines such as IFN-g and IL-2. The study also evaluated the activity of these CAR-T cells *in vivo* following the formation of tumors overexpressing LMP1 in mice. Anti-EBV CAR-T cells were injected intra-tumorally and led to a reduction in tumor growth. However, it is known that a great amount of LMP1 is located in endosomes, which decreases its accessibility on the surface of EBV-associated tumor cells. Consequently, CAR T cells targeting LMP1 act in a level below their potential [50], although Tang *et al*. [51] also showed that neural progenitor cells overexpressing LMP1 were killed by the anti-EBV lymphocytes. It is still a challenge to reproduce the same outcomes considering the physiological levels of LMP1 and the respective cytotoxicity potential of T cells carrying the CARs. Nonetheless, these data show that this approach is promising and deserves further development for EBV-associated malignancies.

Another recent attempt of bringing CAR-T technology into the infectious diseases setting was the development of a CAR-T cell to fight opportunistic *Aspergillus* fungal infections, which poses a real hazard to immunocompromised patients. Kumaresan *et al*. [52] designed a CAR based on the pattern recognition receptor Dectin-1 that recognizes β-glucans present in the fungi cell wall. The so-called ‘D-CAR’ (Fig. 3E) comprising the extracellular portion of Dectin-1, the CD28 costimulatory domain, and the CD3z chain was able to reduce hyphal growth *in vitro* and lead to a decrease in fungal burden on *in vivo* NOD SCID-γ (NSG) mice models of lung and cutaneous *Aspergillus* infection. Despite being an interesting proof-of-concept, it has been shown that Dectin-1 can bind ligands other than β-glucans and may even serve as a co-stimulus when binding to CD4 and CD8 T cells [53, 54]. This lack of specificity is daunting as regards to translating this approach to the clinic.

Despite the great potential of using CAR-T cells in infectious diseases, it must be considered that vital organs are involved, such as the liver in different types of Hepatitis and therefore T cells can both induce remission of the disease or damage as in the case of cytokine release syndrome. Thus, the use of CAR-T cells should be carefully assessed for each type of disease.

One of the alternatives to solve this issue would be to use suicide genes to eliminate the CAR-T cells, or even to bet on the transient expression of these CARs using, for example, the electroporation of CAR mRNA. By doing so, it would be possible to monitor and transfer in a staggered manner the ideal number of T cells to patients and monitor their toxic effects [45].

### CAR-T cells in autoimmune diseases

Systemic lupus erythematosus (SLE) is an autoimmune disease characterized by multiple organ systems involvement, where autoantibodies against DNA and nuclear proteins form immune complex deposits consistently linked to disease pathogenesis [55]. Rituximab, an anti-CD20 monoclonal antibody, has been tried clinically in lupus nephritis, but failed to induce durable remissions [56, 57]. It has been hypothesized that immune complexes could block macrophage phagocytosis, and therefore, Rituximab doses need to be higher [58, 59]. Furthermore, incomplete B cell depletion and the dosing schedule may have contributed to the lack of success. Alternatively, CD19-targeted CAR-T cells are capable of inducing long lasting and profound B cell depletion through direct cytotoxicity, posing an attractive option for lupus treatment [60].

**Figure 4.**
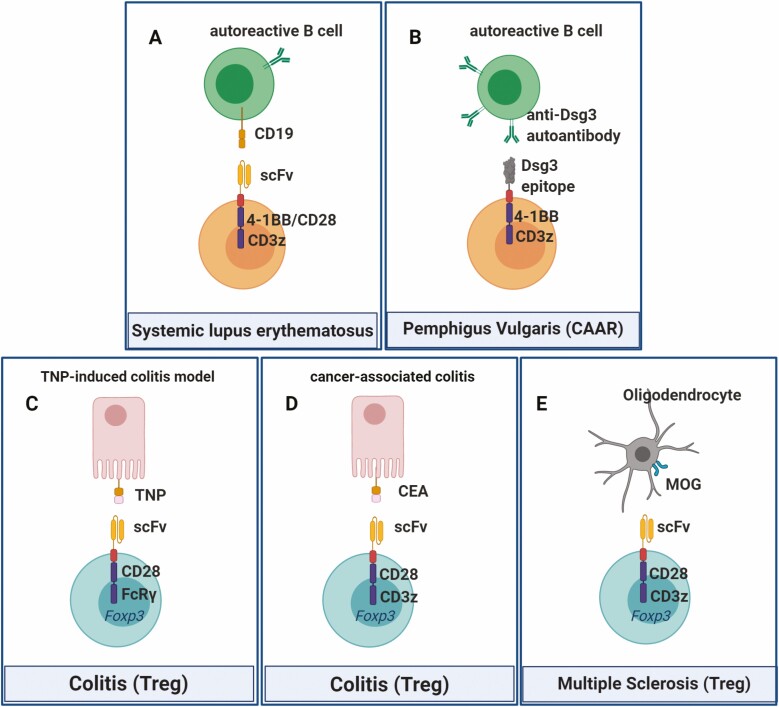
CAR-T cells for autoimmune diseases. (A) Second-generation CAR with scFv domain targeting CD19 for B cell depletion in SLE. (B) CAAR expressing extracellular cadherin domains of Dsg3 recognizes BCRs anti-Dsg3, leading to autoreactive memory B cells elimination in PV. (C) Tregs transduced with anti-TNP CAR suppressed inflammation and improved survival in a murine model of TNP-induced colitis. (D) Similarly, Tregs expressing anti-carcinoembryonic antigen CAR reduced the severity of colitis and subsequent colorectal tumor burden in two types of induced colitis in mice. (E) Intranasally delivered anti-myelin oligodendrocyte glycoprotein CAR Tregs reached various brain areas and suppressed inflammation, leading to clinical improvement in a MS murine model.

Indeed, Kansal *et al*. showed that CD19-targeted CD8+CAR-T cells were capable of reversing or deferring lupus manifestations in two murine models (NBZ/W and MRL-lpr), with considerable extension of the animals’ lifespan [60]. Because auto reactive CD4+ T cells might enhance disease, CD8+ T cells were purified and used to express a second-generation CAR, with the extracellular single-chain Fv domain of an anti-CD19 antibody, linked to CD28 and a variant CD3z terminus. Jin *et al*. further corroborated these findings using the MRL-lpr lupus model and a similar CAR-T configuration. However, in this study, a second construct was added for comparison, which used 41BB instead of CD28 costimulatory domain (Fig. 4A). Profound and sustained B cell depletion was achieved and correlated with amelioration of lupus symptoms [61]. The 41BB construct had better therapeutic effects compared to the one with CD28, which is consistent with previous findings that show longer persistence and lower exhaustion levels with 41BB signaling domain [62, 63]. The results displayed are encouraging for further research in lupus, especially since anti-CD19 CAR-T cells are already licensed for clinical use. Indeed, CD19 CAR-T cells for SLE have recently moved into a phase I clinical study (NCT03030976). Following a similar rationale, anti-B cell maturation antigen (BCMA) CAR-T cells initially developed against multiple myeloma [64, 65] are currently in a phase I/II clinical study (NCT04146051) using mRNA-transfected T cells to treat myasthenia gravis, an autoimmune disease in which B cells target the acetylcholine receptor in neuromuscular junctions.

A novel approach to target autoimmunity was used for pemphigus vulgaris (PV), a life-threatening autoimmune disorder characterized by disseminated skin and mucosal blistering that can lead to fatal infections [66]. PV is caused by autoantibodies against Dsg3 (Desmoglein-3) and although CD20-targeted therapy induces remission in 95% of patients, most of them will relapse [67]. During active disease and relapse, the same anti-Dsg3 B cell clones are observed. Therefore, elimination of those memory cells could lead to PV cure. Ellebrecht *et al*. created a chimeric auto-antibody receptor (CAAR) [66], which comprised the cadherin domains 1–4 of Dsg3 as extracellular domains, coupled to the CD8 transmembrane domain linked to 41BB costimulatory and CD3z signaling domains (Fig. 4B). The authors were also able to observe, in a murine model of PV, that these CAAR-T cells were capable of selectively eliminating anti-Dsg3 cells without off-target related toxicity [66]. There is a phase I clinical trial (NCT04422912) ongoing for this disease aiming to evaluate the maximum tolerated dose and optimal infusion schedule of Dsg3-CAART cells in mucosal-dominant PV.

Yet another alternative in the context of autoimmune diseases is the introduction of CARs into regulatory T cells (Treg), generating antigen specific Treg cells. Infusion of polyclonal Treg cells has been used in phase I trials to halter type I diabetes and graft-versus-host-disease, with limited success and good safety profile [39]. Designing an antigen-specific Treg would allow for lower doses necessary to induce immunosuppression, and the possibility to redirect Treg cells to specific tissues, minimizing the off-target effects. A few models have been described in this scenario. In one of the first descriptions, by Elinav *et al*. [68], using a murine 2,4,6-Trinitrophenol (TNP)-induced colitis model, Treg cells were transduced with a second-generation CAR comprising a scFv specific for TNP (Fig. 4C). After retroviral transduction, Tregs maintained high Foxp3 expression and showed antigen-specific suppression activity *in vitro*, while significantly alleviating symptoms of TNP-induced colitis *in vivo*. A subsequent report by Blat *et al*. [69] using two different models of murine colitis (T-cell- and chemically induced) also suggests good potential for clinical use of CAR Treg for ulcerative colitis amelioration and colon cancer hindering. The designed CAR model targeted the membrane proximal A3 domain of the carcinoembryonic antigen molecule (Fig. 4D), which is overexpressed in colitis and colon cancer, leading to significantly reduced inflammation and lower tumor burden.

Multiple sclerosis (MS), an autoimmune demyelinating disease that affects the central nervous system (CNS) and leads to significant morbidity, has also been subject of interest in CAR-T therapy development. In a murine experimental autoimmune encephalomyelitis (EAE) model of MS, CNS-targeted CAR-Treg cells were effective in suppressing inflammation and reducing symptoms [70]. Interestingly, the CAR construct CARαMOG-FoxP3, which included a scFv anti-rat myelin oligodendrocyte glycoprotein (Fig. 4E), was also coupled *in trans* to the murine FoxP3 gene, separated by a 2A peptide. Forcing FoxP3 in this strategy aims to lock T cells in the regulatory phenotype, avoiding plasticity-derived phenotypes that could drive the cells toward a proinflammatory profile. A lentiviral vector was used to transduce murine naive CD4 cells, which were later delivered intranasally to treat EAE. CAR-Treg cells could reach various brain areas and suppress inflammation, as demonstrated by reduced levels of IFN-g mRNA in the brain and decreased demyelination as assessed by immunohistochemical markers. The benefits persisted even after rechallenging with a second EAE-inducing inoculum, showing the persistence of cells. Very importantly, the Treg cells retained their suppressive phenotype even after challenged with lipopolysaccharides (LPS)-stimulated macrophages, thought to reverse the Treg phenotype into an effector one. These findings show a promising new treatment strategy for MS and warrant further studies [70].

The main concern regarding the Treg strategy is the possibility that the inflammatory milieu could convert the Treg cells into T effector cells, with catastrophic consequences [71]. A few strategies have been proposed to overcome this issue, such as good manufacturing practices quality control using Treg cell-specific demethylated region as a marker for effector T cell conversion potential [72]. Suicide switches linked to Foxp3 inactivation or inflammatory cytokines expression are also envisioned [73]. Another strategy regards modulation of signal strength, since Tregs demand lower TCR affinity for activation [74]. Although promising, further investigation of this aspect is paramount for safe clinical use of CAR Treg cells.

### CAR-T cells in fibrotic diseases: emerging applications

**Figure 5.**
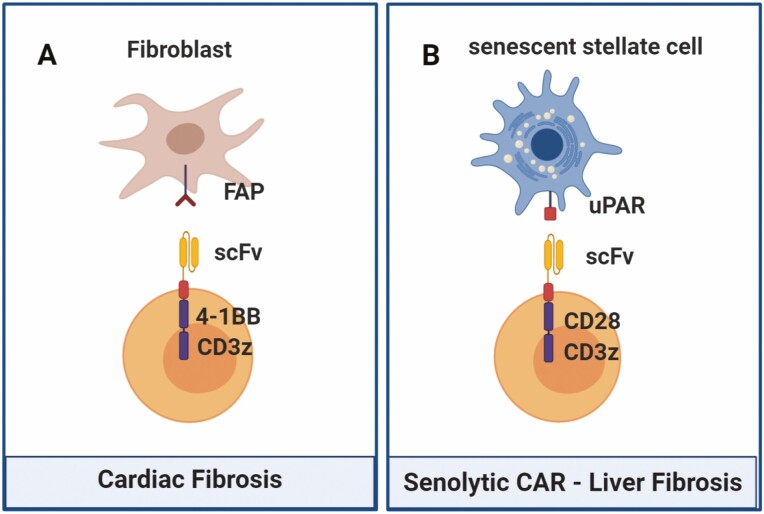
CAR-T cells for cardiac fibrosis and senescence-associated liver fibrosis. (A) Anti-FAP second-generation CAR-T cells were recently proposed as a treatment strategy for cardiac fibrosis, as FAP is highly expressed in cardiac fibroblasts. (B) A novel approach using a uPAR-targeting CAR brings to light the possibility of driving T cells against senescent cells present in several diseases, specifically liver fibrosis, in which senescent hepatic stellate cells play an important role.

Cardiac diseases remain one of the leading mortality causes in the world [75]. Most myocardial diseases lead to cardiac fibrosis, which diminishes organ function and contributes to progression of heart failure [76]. Fibrosis reversing therapies are extremely limited and novel treatment strategies are needed. Recently, Aghajanian *et al*. identified an endogenous target for cardiac fibroblasts – the fibroblast activation protein (FAP) – through gene signature analysis of healthy and diseased human hearts [76]. In a murine model of cardiac injury induced by angiotensin II and phenylephrine, a FAP-targeted CD8 CAR-T cell (Fig. 5A) was capable of significantly reducing fibrosis and restoring cardiac function post-injury. Interestingly, CAR-T cells targeting FAP were previously described in 2013 by Tran *et al*. [77] to target solid tumors. As stromal fibroblasts are highly present in the tumor microenvironment and contribute to progression and metastasis, FAP-expressing stromal cells were an attractive target for CAR-based therapy. However, third-generation FAP-targeting CAR-T cells in this study showed limited antitumor activity and induction of cachexia and other toxicities, which were attributed to the expression of FAP on bone marrow stromal cells that could also be recognized in an on-target/off-tumor fashion [77]. Nonetheless, shortly after this first study, three other reports using FAP-targeting CAR-T cells were published that contrasted the previous results. Kakarla *et al*. [78] used an anti-FAP CAR specific for both murine and human FAP with a CD28 costimulatory domain against murine lung cancer models and saw a decrease in tumor growth that could be further enhanced by co-treatment with tumor-targeting cells. Accordingly, Schubert *et al*. [79] also reported *in vitro* and *in vivo* functionality of anti-FAP CAR-T cells in a mesothelioma xenograft model. In an additional example, Wang *et al*. [80] developed a second-generation CAR with the 41BB activation domain specific for murine FAP and tested it against mesothelioma and lung cancer mouse models. The authors reported that the CAR-T cells showed specific cytotoxicity against FAP+ cells that resulted in inhibited tumor growth. Importantly, both Kakarla *et al*. and Wang *et al*. reported no clinical toxicity. The reasons behind the different toxic effects seen in these studies are still not clear.

In the study by Aghajanian *et al*. targeting cardiac fibrosis, no off-target effect was observed in non-cardiac organs, neither delay in wound healing. Notably, perivascular fibrosis persisted, consistent with the absence of FAP expression in perivascular fibroblasts. Overall, it seems that the anti-FAP CAR approach has good potential for clinical application both for malignant and non-malignant conditions. Considering the prevalence of cardiac disease, notably the ischemic type, in the population, the development of such an approach is of great need.

Recently, great attention has been brought by the possibility of using CAR-T cells to target cellular senescence and reversing senescence-associated pathologies, as shown by Amor *et al*. [81]. Cellular senescence is a well-characterized state consisting of stable cell cycle arrest and a secretory program that modulates the tissue microenvironment [82]. Senescence is a physiological process that prevents pre-malignant cells proliferation and promotes wound healing. However, pathological accumulation of senescent cells associated with secreted pro-inflammatory factors can lead to tissue inflammation and damage, which empowers progression of degenerative diseases, such as atherosclerosis, diabetes, osteoarthritis, lung, and liver fibrosis. Through gene expression analysis of three distinct senescence models, urokinase-type plasminogen activator (uPAR) was identified as a cell-surface protein consistently and specifically upregulated in senescent cells. A second-generation uPAR-specific CAR was constructed (Fig. 5B), using an anti-mouse uPAR single-chain variable fragment linked to the CD28 costimulatory molecule and the CD3z signaling domain [81]. Those CAR-T cells were effective in ablating senescent cells *in vitro* and *in vivo* using murine models of lung adenocarcinoma treated with senescence inducing drugs and two models of liver fibrosis (chemical- and diet-induced), in which senescent hepatic stellate cells contribute to disease progression. Elimination of senescent cells translated into improved survival in a lung cancer model, and restored liver function, without notable toxicities in the proposed doses. Even though it is quite early for clinical application and further studies to address safety are needed, this model shows that senolytic CAR-T cells may be a feasible treatment for several senescence-associated pathologies.

## Conclusion and perspectives

In the last decade, enormous advances have been achieved with anti-tumor CAR-T cell therapy, most notably in fighting hematological malignancies. However, CAR-T technology has great potential for broad clinical use in the non-malignant setting as well, especially for infectious and immune-mediated diseases. As discussed in this review, even though CAR-T cell therapy for HIV cure so far has been unsuccessful, several new concepts have been developed which deserve further exploration. Besides HIV, CAR-T cells targeted against HBV, HCV, CMV, EBV, and *Aspergillus* have been designed but are still in an early preclinical stage, with limited efficacy demonstrated so far. CAR-T development for other infectious diseases like tuberculosis has been proposed [83], but its feasibility is yet to be determined. For autoimmune diseases, CAR-T cell therapy brings a palpable opportunity for ameliorating or even curing a variety of chronic and degenerative diseases, with ongoing clinical trials. Furthermore, with the recently highlighted ability to target pathological fibrosis and cellular senescence, two processes deeply connected to chronic inflammation and cancer, the horizon of possibilities gets even wider.

## Data Availability

No new data were generated or analyzed in support of this research.

## Abbreviations

BLT: Bone marrow, liver, and thymus
BCMA: B cell maturation antigen
bNAbs: Broadly neutralizing antibodies
CAR: Chimeric antigen receptor
CAAR: Chimeric auto-antibody receptor
cART: Combination antiretroviral therapy
CMV: Cytomegalovirus
CNS: Central nervous system
CTLs: Cytotoxic T lymphocytes
CXCR5: CX chemokine receptor 5
CXCL13: CXC chemokine ligand 13
Dsg3: Desmoglein 3
EAE: Encephalomyelitis
EBV: Epstein-Barr virus
Env: Envelope protein
FAP: Fibroblast activation protein
HDR: Homology-directed repair
HIV: Human immunodeficiency virus
HSPC: Hematopoietic stem and progenitor cells
HCV: Hepatitis C virus
HBV: Hepatitis B virus
LMP1: EBV latent membrane protein 1
LRA: Latency reversing agent
MS: Multiple sclerosis
NSG: NOD Scid-γ
PV: Pemphigus vulgaris
scFv: Single-chain variable fragment
SLE: Systemic lupus erythematosus
Tfh: Follicular helper T cells
TNP: 2,4,6-Trinitrophenol
Treg: Regulatory T cell
uPAR: Urokinase-type plasminogen activator
